# Screening of pathologically significant diagnostic biomarkers in tears of thyroid eye disease based on bioinformatic analysis and machine learning

**DOI:** 10.3389/fcell.2024.1486170

**Published:** 2024-10-30

**Authors:** Xingyi Shu, Chengcheng Zeng, Yanfei Zhu, Yuqing Chen, Xiao Huang, Ruili Wei

**Affiliations:** Department of Ophthalmology, Changzheng Hospital of Naval Medical University, Shanghai, China

**Keywords:** thyroid eye disease, secretory proteins, lacrimal gland, immune infiltration, machine learning

## Abstract

**Background:**

Lacrimal gland enlargement is a common pathological change in patients with thyroid eye disease (TED). Tear fluid has emerged as a new source of diagnostic biomarkers, but tear-based diagnostic biomarkers for TED with high efficacy are still lacking.

**Objective:**

We aim to investigate genes associated with TED-associated lacrimal gland lesions. Additionally, we seek to identify potential biomarkers for diagnosing TED in tear fluid.

**Methods:**

We obtained two expression profiling datasets related to TED lacrimal gland samples from the Gene Expression Omnibus (GEO). Subsequently, we combined the two separate datasets and conducted differential gene expression analysis and weighted gene co-expression network analysis (WGCNA) on the obtained integrated dataset. The genes were employed for Gene Ontology (GO) enrichment analysis and Kyoto Encyclopedia of Genes and Genomes (KEGG) pathway analysis. The genes were intersected with the secretory proteins profile to get the potential proteins in the tear fluid. Machine learning techniques were then employed to identify optimal biomarkers and develop a diagnostic nomogram for predicting TED. Finally, gene set enrichment analysis (GSEA) and immune infiltration analysis were conducted on screened hub genes to further elucidate their potential mechanisms in TED.

**Results:**

In our analysis of the integrated TED dataset, we identified 2,918 key module genes and 157 differentially expressed genes and finally obtained 84 lacrimal-associated key genes. Enrichment analysis disclosed that these 84 genes primarily pertain to endoplasmic reticulum organization. After intersecting with the secretory proteins, 13 lacrimal gland-associated secretory protein genes (LaSGs) were identified. The results from machine learning indicated the substantial diagnostic value of dyslexia associated gene (KIAA0319) and peroxiredoxin4 (PRDX4) in TED-associated lacrimal gland lesions. The two hub genes were chosen as candidate biomarkers in tear fluid and employed to establish a diagnostic nomogram. Furthermore, single-gene GSEA results and immune cell infiltration analysis unveiled immune dysregulation in the lacrimal gland of TED, with KIAA0319 and PRDX4 showing significant associations with infiltrating immune cells.

**Conclusions:**

We uncovered the distinct pathophysiology of TED-associated lacrimal gland enlargement compared to TED-associated orbital adipose tissue enlargement. We have demonstrated the endoplasmic reticulum-related pathways involved in TED-associated lacrimal gland lesions and established a diagnostic nomogram for TED utilizing KIAA0319 and PRDX4 through integrated bioinformatics analysis. This contribution offers novel insights for non-invasive, prospective diagnostic approaches in the context of TED.

## 1 Introduction

Thyroid eye disease (TED), also referred to as thyroid-associated ophthalmopathy (TAO), thyroid orbitopathy, or Graves’ ophthalmopathy (GO), is a disfiguring autoimmune inflammatory disease affecting the orbital tissues and is usually regarded as an extrathyroidal manifestation of Graves’ disease (GD), which is the leading cause of hyperthyroidism (Bahn, 2010; Bartalena et al., 2021). TED occurs in 25%–50% of individuals diagnosed with GD (Chen et al., 2019). GD stems from the loss of immunotolerance to autoantigen thyrotropin receptor (TSHR) and the subsequent production of thyrotropin receptor antibody (TRAb). Orbital fibroblasts (OFs) express TSHR and can be activated by TRAb as well (Smith and Hegedus, 2016). Upon activation, OFs differentiate into myofibroblasts and adipocytes and eventually lead to orbital adipose tissue (OAT) enlargement and extraocular muscle fibrosis (Bahn, 2010). TED patients manifest ranging from eyelid edema, chemosis, eyelid retraction, lid lag of the upper eyelid on downgaze, exophthalmos, diplopia, restrictive extraocular motility, exposure keratopathy to dysthyroid optic neuropathy (DON), which may incur eyesight loss in severe cases. Consequently, though TED is relatively rare (estimated incidence:1.9 cases per 10,000 population per year) (Hoang et al., 2022), it seriously affects the quality of life (QoL) of patients and increases the risk of anxiety, depression and cognitive impairment evidenced by functional magnetic resonance imaging (Qi et al., 2021; Jiang et al., 2023; Luo et al., 2024).

Early diagnosis and treatment are crucial to improve prognosis and QoL. Mild TED can progress to more severe disease when early diagnosis and treatment are absent (Wiersinga et al., 2018). In addition, available treatments for TED remain lacking. The cumulative dose of intravenous glucocorticoid, the first-line therapy for moderate-to-severe TED patients within the active phase, can only accelerate the course of the disease into the stable phase but hardly improves proptosis (Bartalena and Tanda, 2022). TED diagnosis relies highly on subjective ophthalmological examinations and thyroid serology according to current diagnosis criteria (Bartley and Gorman, 1995). However, ophthalmological examinations carried out by specialized ophthalmologists are largely subjective. Serological tests suggesting hyperthyroidism are significant in the diagnosis of TED, but there exist about 10% of TED patients hypothyroid or euthyroid that are prone to be overlooked (Marcocci et al., 1989). Moreover, thyroid function tests are invasive and these indicators are not parallel with TED severity (Bartalena et al., 2021). Serum antibodies to TSHR elevation are one of the characteristics of GD. The same unequivocal diagnostic correlation does not apply to TED, as numerous patients with elevated TRAb do not develop orbitopathy (Ueland et al., 2023). Therefore, seeking new biomarkers with high diagnostic efficacy is of great significance for the early screening of TED patients and the improvement of their prognosis.

Given that tear fluid is a complex mixture of proteins, lipids, carbohydrates, metabolites, electrolytes, and salts (Gijs et al., 2023), which can offer extensive information, tears have been emerging as the new source of biomarkers for disease diagnosis in recent years such as diabetes mellitus, dry eye disease (DED), uveitis, glaucoma and Alzheimer’s disease, etc. (Burgos-Blasco et al., 2020; Iyengar et al., 2020; Garcia-Onrubia et al., 2022; Kumar et al., 2023; Lee et al., 2023) Tear collection is non-invasive and not restricted to particular operators and instruments so tear sample testing is beneficial to continuous and point-of-care (POC) monitoring for some chronic diseases. The tear film is structured in three distinct layers, from the inner to the outer surface: the mucin layer, the aqueous layer, and the lipid layer. These layers collectively and synergistically maintain the homeostasis of the ocular surface, providing comprehensive protection (Masoudi, 2022). The intermediate aqueous layer is primarily secreted by the lacrimal glands which have been reported to be altered structurally and functionally in patients with TED evidenced by multidetector computed tomography (MDCT) and tear film break-up patterns (Ishikawa et al., 2019; Takahashi et al., 2021; Starcevic et al., 2023). Enlargement of the lacrimal gland is positively correlated with the subjective experience of tearing and a concomitant decline in QoL among patients suffering from TED (Ugradar et al., 2023).

Tear film break-up patterns indicate TED more frequently induces aqueous-deficient dry eye, potentially due to the expression of TSHR in the lacrimal glands (Eckstein et al., 2004). Another study indicated a significant correlation between the size of the lacrimal gland and the concentrations of IL-1β, IL-17A, and IL-6 in tears of TED patients. This correlation suggests that elevated levels of inflammatory cytokines are pivotal in the pathogenesis of ocular surface damage of TED and may be linked to the inflammatory processes affecting the lacrimal gland (Huang et al., 2014). Several studies have shown the value of tears in the diagnosis and monitoring of TED. A study included 18 healthy people and 54 patients with autoimmune thyroid disease (AITD). Among the patients with AITD, 18 had mild TED and 18 had moderate to severe TED. This study found that calcium-binding protein A4 (S100A4) and prolactin-induced protein (PIP) in tears were associated with the occurrence and severity of TED (Chng et al., 2018). A previous study of our department analyzed the proteomics of 30 TED cases and 30 healthy subjects by liquid mass spectrometry and screened for 103 differentially expressed proteins (DEPs), which were mainly enriched in immune-related complement activation and coagulation cascade pathways as revealed by KEGG analysis (Zhou et al., 2022). However, considering that exophthalmos-induced DED always overlaps with TED, screening for biomarkers of TED in tears may be not specific enough. Moreover, these screened DEPs may fail to explain the pathology of lacrimal glands in TED.

This study employed various integrative bioinformatics tools to identify hub genes and explore the potential mechanisms underlying TED-associated lacrimal gland dysfunction by analyzing two TED datasets from the Gene Expression Omnibus (GEO) database. Additionally, four machine learning methods were applied to establish a diagnostic nomogram model for predicting TED based on the screened hub genes, KIAA0319 and PRDX4, which were associated with lacrimal dysfunction. Finally, the CIBERSORT method was used to reveal the relationship between the hub genes and the immunological landscape. The present work may promote the understanding of the pathological mechanism of TED-associated lacrimal gland lesions and reveal a new non-invasion strategy based on a tear fluid test to diagnose TED.

## 2 Methods

### 2.1 Microarray data acquisition and pre-processing

The raw expression profile datasets for TED and the control group, specifically GSE588331 and GSE105149, were downloaded from the Gene Expression Omnibus (GEO) database (Home - GEO - NCBI (nih.gov)). The GSE58331 dataset contains both lacrimal glands and anterior orbital adipose tissue from TED patients and control subjects. Detailed descriptive information on these two datasets is provided in Table 1. The integrated TED expression data was generated by the batch correction to the two TED datasets using the combat function of the “SVA” package (Leek et al., 2012) within R software (version 4.3.2). The final integrated dataset contained 12 TED lacrimal gland samples and 14 control lacrimal gland samples.

**TABLE 1:** Characteristics of TED-associated datasets in GEO.

GEO accession	Platform	Origin	Sample		Species
			TED	Normal	
GSE58331	GPL570	Lacrimal gland	8	7	*Homo sapiens*
GSE105149	GPL570	Lacrimal gland	4	7	*Homo sapiens*
GSE58331	GPL570	Anterior orbit	27	21	*Homo sapiens*

### 2.2 Differentially expressed genes (DEGs) analysis

Background correction, normalization and gene symbol conversion were applied to the integrated TED dataset (GSE58331 and GSE105149). DEGs in TED datasets were then identified using the “Limma” package (Ritchie et al., 2015) in R software, employing a threshold of adjusted *P*-value <0.05 and |log(FC)| > 1. The expression patterns of DEGs were subsequently visualized as volcano plots and heatmaps, utilizing the “ggplot2” and “pheatmap” packages in R software, respectively.

### 2.3 Weighted gene co-expression network analysis (WGCNA) and identification of key modules

Given the interconnection of gene sets and the relationship between gene sets and phenotypes, WGCNA was employed to identify highly synergistic gene sets and potential biomarkers. The “WGCNA” package (Langfelder and Horvath, 2008) in R was used to construct co-expression gene networks for the integrated dataset. Initially, the median absolute deviation (MAD) was calculated for each gene, and the top 50% of genes with the lowest MAD were excluded. Then, the “goodSamplesGenes” function in the “WGCNA” package was utilized to detect missing entries, low-weight items, and zero-variance genes. After that, a scale-free co-expression gene network was constructed and a soft threshold power (β) was set to 22 based on gene correlations. The adjacency was then transformed into a topological overlap matrix (TOM) to measure the network connectivity, defined as the sum of a gene’s adjacency with all other genes. The corresponding dissimilarity (1-TOM) was calculated as well. Genes with similar expression profiles were grouped into gene modules using average linkage hierarchical clustering according to TOM-based dissimilarity measure, with a minimum size of 30 for the genes dendrogram and the sensitivity set to 3. To refine the module analysis, the dissimilarity of module eigengenes was computed, and a cut-off line of 0.25 was applied to merge similar modules. Finally, the module-feature matrix and sample information matrix were calculated, and their correlation was visualized using the “labeledHeatmap” function.

### 2.4 Establishing lacrimal-associated key genes

Jvenn (Bardou et al., 2014) (jvenn (inra.fr)) was used to identify the lacrimal-associated key genes of TED. By inputting the DEGs and genes from the key modules obtained through WGCNA, the lacrimal-associated key genes were determined and the results were visualized by Venn diagrams.

### 2.5 Secretory proteins access and intersection with lacrimal-associated key genes

Secretory proteins were obtained from The Human Protein Atlas (HPA) database (The Human Protein Atlas). A total of 3,970 genes coding secretory proteins were downloaded from the protein class of “SPOCTOPUS predicted secreted proteins” (Search: protein_class: SPOCTOPUS predicted secreted proteins - The Human Protein Atlas). Jvenn was used to intersect the secretory proteins with the lacrimal-associated key genes, identifying lacrimal-associated key secretory genes (LaSGs).

### 2.6 Functional enrichment analysis

For functional enrichment analysis, we utilized GO (Gene ontology) and KEGG (Kyoto Encyclopedia of Genes and Genomes) annotations from the R package “org.Hs.eg.db”, and performed the enrichment analysis using the R package “clusterProfiler” (version 3.14.3) to. The gene set size was restricted to a minimum of 5 and a maximum of 5,000. *P*-value <0.05 was considered statistically significant. The results of the enrichment analysis were visualized employing the “ggplot2” and “enrichplot” packages.

### 2.7 Analysis of the GeneMANIA database

To investigate the interactions among LaSGs, we constructed a gene-gene interaction network using the GeneMANIA database (GeneMANIA). The network was based on physical interactions, co-expression, and co-localization among the LaSGs.

### 2.8 Machine learning algorithms

To identify potential tear biomarkers and develop a diagnostic model for TED, the LaSGs were first subjected to the least absolute shrinkage and selection operator (LASSO) regression analysis (Antonacci et al., 2019) using the “glmnet” package. Next, the “randomForest” package was employed to analyze the LaSGs using the random forest (RF) algorithm (Savargiv et al., 2021), where genes with MeanDecreaseGini values greater than 0.5 in the RF model were defined as candidate hub genes. The “e1071” package in R was used to carry out the support vector machine recursive feature elimination (SVM-RFE) algorithm (Sanz et al., 2018), and the result was assessed using the 5-fold cross-validation error. To further refine the candidate biomarkers, the extreme gradient boosting (XGboost) (Chen and Guestrin, 2016) algorithm was performed using the R packages “XGboost”, retaining genes with importance values greater than 20. Genes that overlapped across the LASSO, RF, SVM-RFE and XGboost models were defined as hub genes for the development of a TED diagnostic model.

### 2.9 Development of a nomogram and assessment of predictive models for diagnostic markers

After machine learning had narrowed down LaSGs, “KIAA0319” and “PRDX4” were selected as hub genes for TED diagnosis. The “rms” package (Harrell, 2017) was utilized to construct a nomogram to visualize the results. Receiver operating characteristic (ROC) curves were generated to evaluate the diagnostic performance of each hub gene and the nomogram in diagnosing TED. Additionally, calibration curves and decision curve analysis (DCA) were employed to further assess the predictive accuracy and efficiency of the nomogram for TED.

### 2.10 Single-gene gene set enrichment analysis (GSEA) analysis

For the GSEA, we downloaded GSEA software (version 3.0) from the official website (DOI:10.1073/pnas.0506580102, http://software.broadinstitute.org/gsea/index.jsp). The samples were divided into groups (≥50%) and low-expression groups (<50%) according to the expression levels of KIAA0319 and PRDX4. We then obtained c5.go.bp.v7.4.symbols.gmt from Molecular Signatures Database (DOI:10.1093/bioinformatics/btr260, http://www.gsea-msigdb.org/gsea/downloads.jsp), which was used to assess gene-associated pathways and molecular functions. *P*-value <0.05 was considered statistically significant.

### 2.11 Immune cell infiltration analysis

Immune infiltration in TED gene expression profile was evaluated using the “CIBERSORT” package (Steen et al., 2020). The abundance and proportions of immune cell infiltration for each sample were visualized through bar plots generated with the “ggplot2” package. The Wilcoxon test was used to compare the proportions of 22 immune cell types between TED and normal samples, with the results presented in the box plot created using “ggplot2”. Furthermore, the correlations among the 22 infiltrating immune cells were illustrated using the “corrplot” package, with *p* < 0.05 considered statistically significant.

## 3 Results

### 3.1 Data acquisition and processing

**FIGURE 1:**
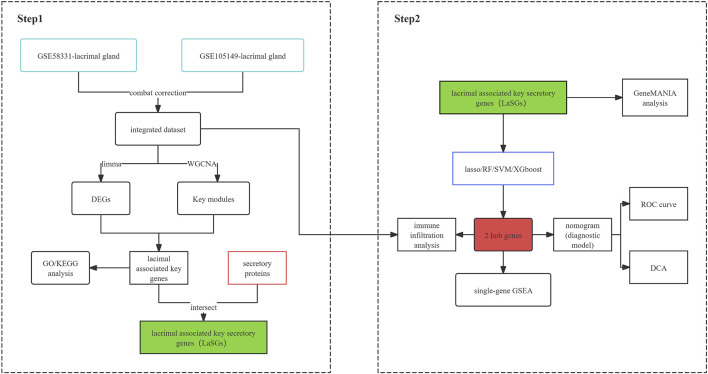
Flow chart of this work.

**FIGURE 2:**
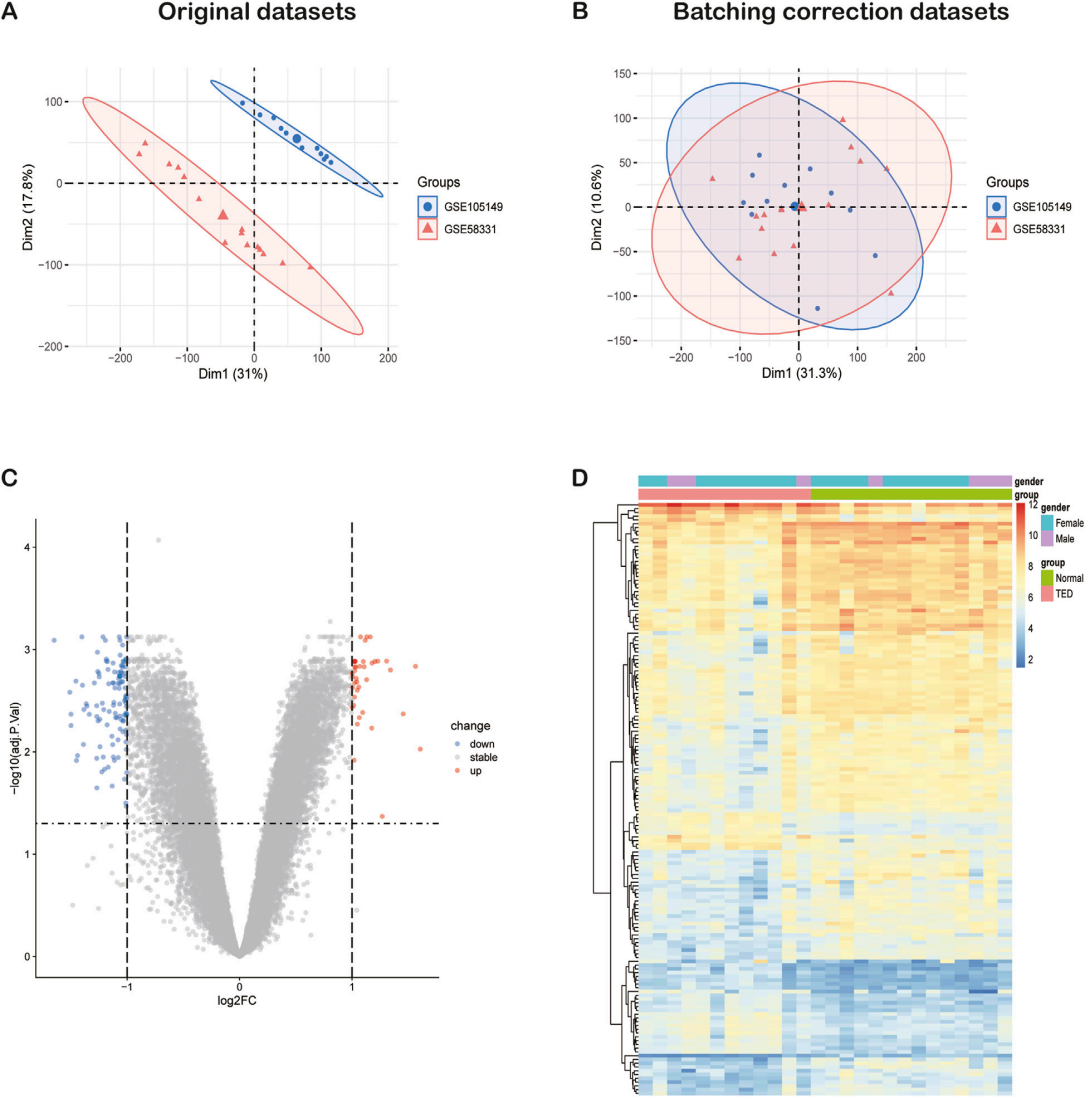
The integration of TED datasets and differential expression analysis of the integrated TED dataset. **(A)** PCA of two original TED datasets before batch-effect correction. **(B)** PCA of the integrated TED dataset after batch-effect correction. **(C)** The volcano plot represents TED DEGs in the integrated TED dataset. The upregulated genes are presented in red dots, whereas, the downregulated genes are presented in blue dots. **(D)** The heatmap shows the upregulated and downregulated DEGs in the integrated TED dataset. TED: thyroid eye disease, PCA: principal component analysis, DEGs: differentially expressed genes.

The general flow chart of this study is shown in Figure 1. Microarray data of lacrimal gland samples from TED and normal control group from two datasets, GSE58331 and GSE105149, were selected for the current study (Table 1), and the two datasets were normalized to eliminate batch effects. Figures 2A, B show a significant reduction in the batch effect between the two datasets. We normalized the data with the batch effect removed, resulting in an integrated dataset containing a total of 12 lacrimal gland samples from TED patients and 14 from normal subjects.

### 3.2 Identification of DEGs in the lacrimal gland of TED

We conducted a DEG analysis on the integrated TED dataset, identifying a total of 157 DEGs, comprising 40 upregulated and 117 downregulated genes when using *p* < 0.05 and |log2 (fold change) | >1 as selection criteria. The expression patterns of these 157 DEGs are visualized in the volcano plot and heatmap (Figures 2C, D). Considering that females are at a higher risk of developing TED (Bahn, 2010), the heatmap also illustrates the relationship between the expression patterns of the 157 DEGs and gender.

### 3.3 Construction of weighted gene co-expression network and extraction of genes in key modules

**FIGURE 3:**
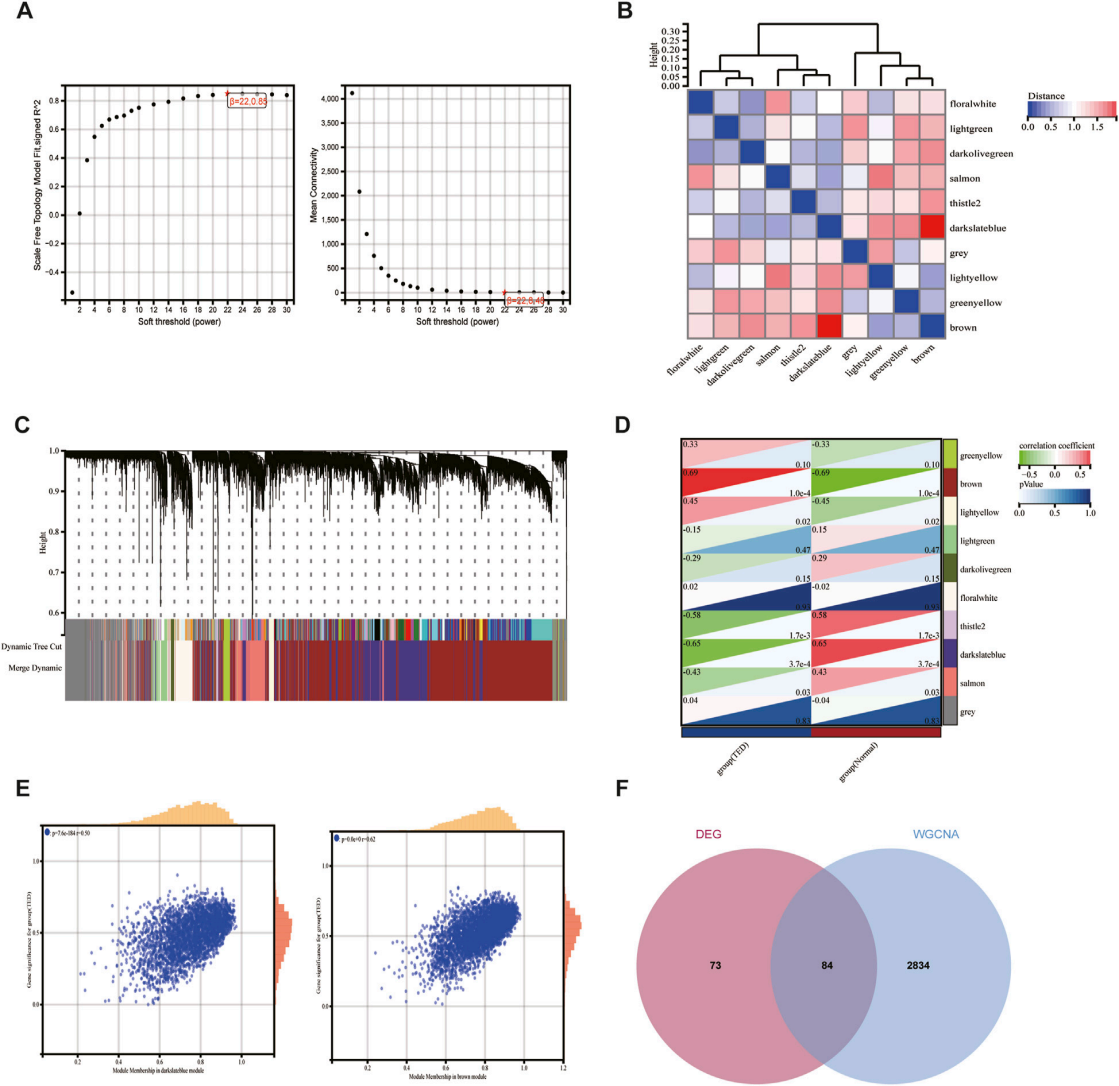
Screening of key module genes in the integrated TED dataset via WGCNA and identification of TED lacrimal gland-associated key genes through the intersection of key module genes and DEGs. **(A)** The scale-free topology model was utilized to identify the best β value, and β = 22 was chosen as the soft threshold based on the average connectivity and scale independence. **(B)** The network heatmap shows the gene dendrogram and module eigengenes. **(C)** The cluster dendrogram presenting module eigengenes. **(D)** The heatmap reveals the relationship between module eigengenes and TED. The correlation coefficient (upper) and *p*-value (bottom) of module eigengenes and TED were presented. The brown and darkslateblue modules correlated with TED and exhibited the highest positive or negative correlation coefficients, respectively, which were identified as the key modules in CAVD. **(E)** The correlation plot between the brown and darkslateblue module membership and the gene significance of genes in the two modules. **(F)** A total of 84 key genes were identified by taking the intersection between key modules’ genes and DEGs via the Venn diagram. WGCNA: weighted gene co-expression network analysis, TED: thyroid eye disease, DEG: differentially expressed genes.

WGCNA facilitates the screening of genes with similar expression patterns and aggregates genes with similar expression patterns into the same module. A soft threshold (β) of 22 was chosen based on scale independence and mean connectivity (Figure 3A). 10 gene expression modules were identified under the condition of β of 22 and marked with different colors. The distances between different modules are shown in Figure 3B, with the darkblue module and the brown module being the most distant from each other and having the lowest correlation. The clustering of module eigengenes is displayed in Figure 3C. Figure 3D demonstrated that the brown module exhibited the highest positive correlation with TED (r = 0.69, *p* = 1.0e−4) and the darkslateblue module displayed the most negative correlation with TED (r = −0.65, *p* = 3.7e−4). Therefore, genes within the brown and darkslateblue modules were screened out and used for subsequent analyses. Further analysis revealed a strong correlation between genes within the brown and darkslateblue modules and gene significance (r = 0.62, *p* = 0; r = 0.50, *p* = 7.6e−184) (Figure 3E). Ultimately, 1836 genes within the brown module and 1,082 genes within the darkslateblue module, a total of 2,918 genes, were extracted. To further identify genes associated with lacrimal gland lesions in TED patients, we took the intersection of the DEGs and the genes obtained from the WGCNA and ended up with 84 lacrimal gland-associated key genes for subsequent analysis (Figure 3F).

### 3.4 Functional enrichment analysis by GO and KEGG analysis

**FIGURE 4:**
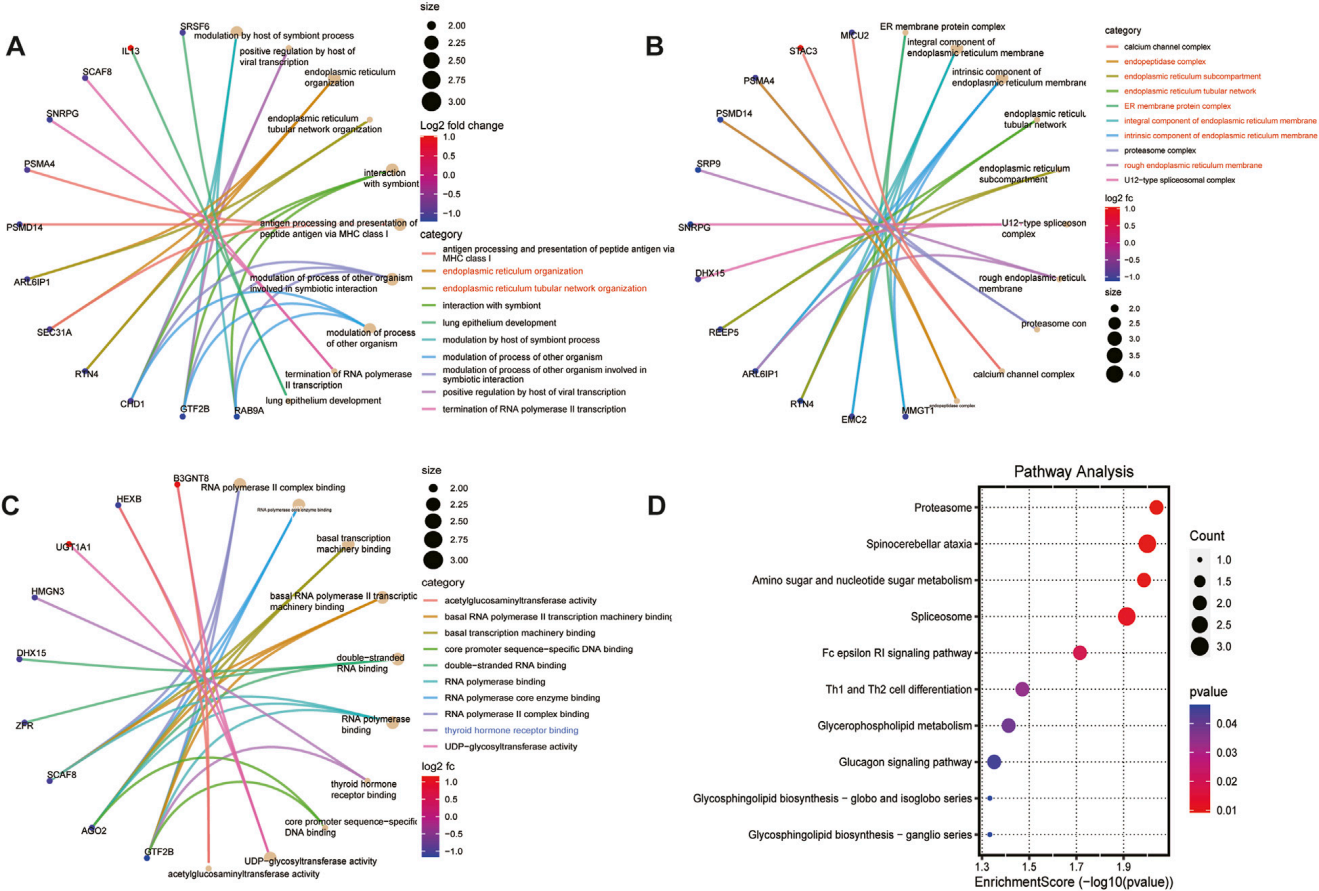
Functional enrichment analysis based on lacrimal gland-associated key genes in lacrimal gland lesions of TED. **(A)** Cnetplot of the results of GO-BP enrichment analysis. **(B)** Cnetplot of the results of GO-CC enrichment analysis. **(C)** Cnetplot of the results of GO-MF enrichment analysis. **(D)** Dotplot of the results of KEGG enrichment analysis. GO: Gene ontology, KEGG: Kyoto Encyclopedia of Genes and Genomes, BP: biological process, CC: cellular component, MF: molecular function.

We further exerted GO and KEGG analysis to deepen our understanding of the functions and specific mechanisms of 84 lacrimal gland-associated key genes in lacrimal gland lesions of TED. Biological process (BP) and Cellular component (CC) of GO term analysis illustrated that the enrichment of the endoplasmic reticulum-related pathways, which were displayed in red in Figures 4A, B. Molecular function (MF) of GO term analysis showed that lacrimal gland-associated key genes in TED were mostly enriched in the polymerase-related pathways such as RNA polymerase II complex binding, RNA polymerase core enzyme binding, basal RNA polymerase II transcription machinery binding, double-stranded RNA binding and RNA polymerase binding (Figure 4C). Furthermore, MF analysis demonstrated the enrichment of the thyroid hormone receptor binding pathway. The KEGG pathway analysis demonstrated a close association between the lacrimal gland-associated key genes and pathways such as “Proteasome”, “Spinocerebellar ataxia”, “Amino sugar and nucleotide sugar metabolism”, “Spliceosome”, “Fc epsilon RI signaling pathway” and “Th1 and Th2 cell differentiation pathway” (Figure 4D).

### 3.5 Identification of lacrimal gland-associated key secretory protein genes and GGI network construction

**FIGURE 5:**
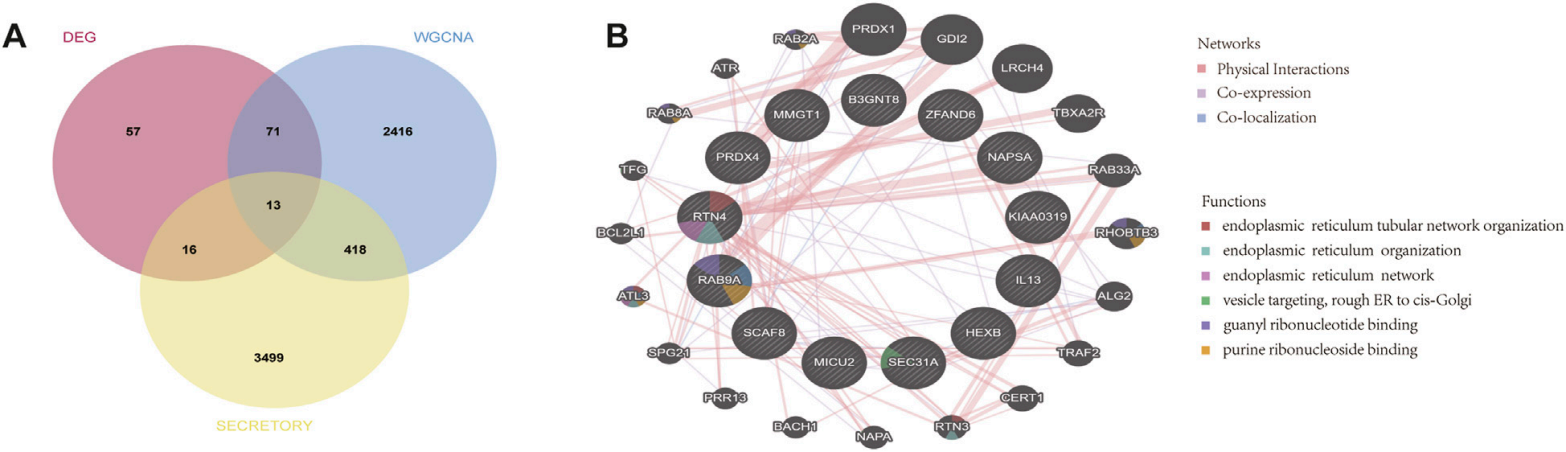
GeneMANIA database analysis and functional enrichment analysis based on LaSGs. **(A)** 13 LaSGs were obtained by the intersection of DEGs, genes in the key modules after WGCNA, and secretory proteins from the HPA. **(B)** GeneMANIA database analysis of CGs, showing a network with a total of 33 genes and 111 connections, including Co-expression, Co-localization and Physical Interactions. LsAGs: lacrimal associated key secretory genes, DEGs: Differentially expressed genes, HPA: Human protein atlas.

Lacrimal gland dysfunction is one of the most important causes of aqueous-deficient dry eye in patients with TED. The aqueous tear fluid secreted by the lacrimal gland is an important component of the tear film (Huang et al., 2014). Identifying differentially expressed lacrimal gland proteins between TED patients and normal subjects and further extracting secreted proteins from them can help to screen TED biomarkers in tear fluid. We took the intersection of 84 lacrimal gland-associated key genes with secretory proteins downloaded from the HPA database and obtained 13 lacrimal gland-associated secretory protein genes (LaSGs) (Figure 5A). Then, we performed GGI network construction and enrichment analysis of these 13 genes using the GeneMANIA online tool (Figure 5B). As illustrated in Figure 4B, the nodes represent both the genes we uploaded and those identified as related through the GeneMANIA search. The lines depict the network categories connecting these genes. Our network comprises 33 genes, including the 11 uploaded genes and 22 associated genes, with a total of 111 interactions, including Co-expression, Co-localization and Physical Interactions. The enrichment analysis revealed that these 33 genes were primarily enriched in the pathways related to endoplasmic reticulum tubular network organization, endoplasmic reticulum organization, endoplasmic reticulum tubular network, vesicle targeting, rough ER to cis-Golgi, guanyl ribonucleoside binding and purine ribonucleoside binding.

### 3.6 Screening for hub genes with diagnostic value via four machine learning methods and construction of a diagnostic model in TED

**FIGURE 6:**
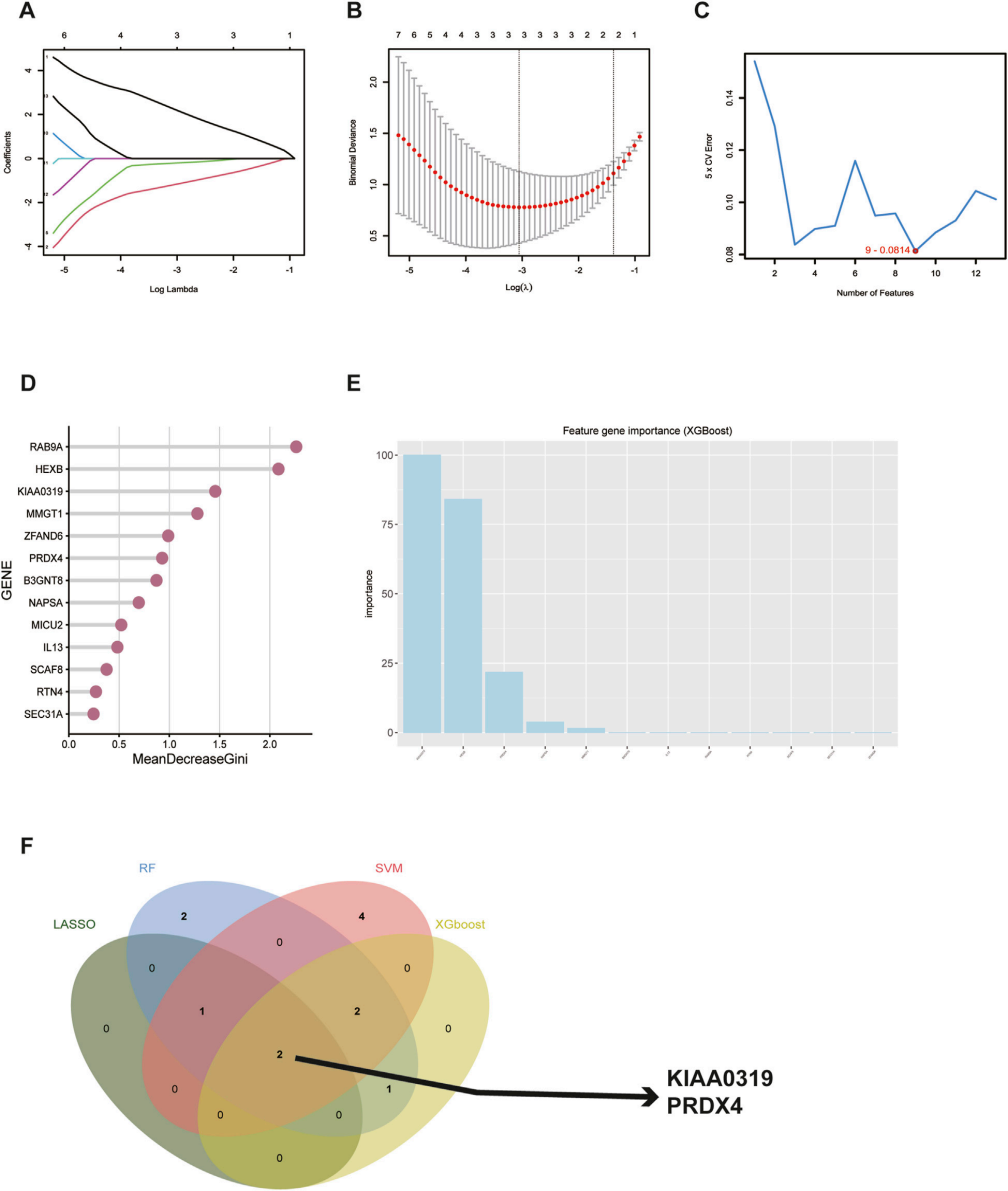
Screening for potential diagnostic biomarkers in tear for TED using machine learning methods. **(A, B)** Lasso regression analysis of the 13 hub genes to calculate the minimum value **(A)** and λ value **(B)** for diagnostic biomarkers. **(C)** Support vector machine recursive feature elimination analysis of the 13 hub genes; selection of 9 biomarkers based on the lowest error. **(D)** Random forest algorithm analysis of the 13 hub genes; selection of biomarkers with MeanDecreaseGini scores greater than 0.5. **(F)** Venn diagram shows the result of the intersection of 4 machine learning methods. TED: thyroid eye disease.

**FIGURE 7:**
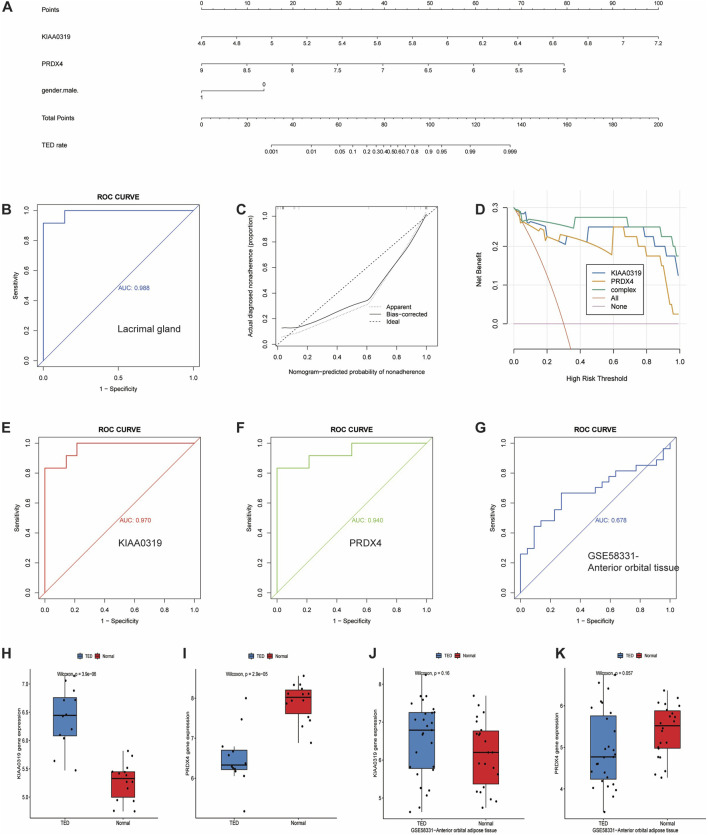
Development and efficacy evaluation of the diagnostic nomogram model. **(A)** Logic regression analysis of gender and 2 genes, including KIAA0319 and PRDX4. **(B)** ROC curve for the diagnostic performance of gender and the two screened biomarkers (KIAA0319 and PRDX4) in the lacrimal gland. **(C)** Calibration curve of the nomogram model predictions in TED, where the dashed line labeled “Ideal” represents the standard curve, representing perfect predictions of the ideal model. The dotted line labeled “Apparent” represents the uncalibrated predicted curve, while the solid line labeled “Bias-corrected” represents the calibrated predicted curve. **(D)** DCA for the nomogram model. The pink line is labeled as “None” representing the net benefit of the assumption that no patients have TED. The orange line is labeled as “All” indicating the net benefit of the assumption that all patients have TED, and the green line is labeled as “complex” representing the net benefit of the assumption that TED cases are identified based on the diagnostic value of TED predicted by the nomogram model. **(E, F)** ROC curve for the diagnostic performance of two screened biomarkers (KIAA0319 and PRDX4) in the lacrimal gland, respectively. **(G)** ROC curve for the diagnostic performance of gender and two screened biomarkers (KIAA0319 and PRDX4) in anterior adipose tissue. **(H, I)** The expression of KIAA0319 and PRDX4 in the lacrimal gland. **(J, K)** The expression of KIAA0319 and PRDX4 in anterior adipose tissue in the GSE58331 dataset. ROC: Receiver operating characteristic, DCA: Decision curve analysis, TED: thyroid eye disease.

To further identify and screen tear biomarkers with high sensitivity and specificity for TED diagnosis, we further downscaled 13 genes using four machine-learning methods. Using LASSO regression, we identified 3 potential candidate genes from the 13 common genes (Figures 6A, B). We applied the SVM model to obtain the correlation coefficients between the 13 genes and TED and applied the 5×crossover operation to derive the lowest error rate of the model when the number of genes was 9 (Figure 6C). Therefore, we screened 9 genes out of 13 genes based on the correlation coefficient. We applied the random forest method and screened 8 potential candidate genes with MeanDecreaseGini >0.5 (Figure 6D). We also used the XGboost method and screened out four candidate genes with the criterion of variable importance>0 (Figure 6E). Finally, we took the intersection of the candidate genes obtained from each of the four machine learning methods and finally obtained two hub genes, KIAA0319 and PRDX4 (Figure 6F). Considering that gender is an important risk factor for a variety of autoimmune diseases, and that the male-to-female sex ratio of those suffering from TED was about 1:4 in previous reports, we constructed a nomogram using the three variables of gender, expression of KIAA0319, and expression of PRDX4 for clinical diagnosis (Figure 7A). We plotted the ROC curve based on the obtained diagnostic model to verify the sensitivity and specificity of the model for the diagnosis of TED and found that the area under the curve (AUC) of the ROC curve of 0.988 (Figure 7B). This indicates the high sensitivity and specificity of the model. The diagnosis of TED using KIAA0319 and PRDX4 as diagnostic markers alone also had high diagnostic efficacy (AUC = 0.970; AUC = 0.940, Figures 7E, F)), but not as high as the diagnostic model constructed based on gender and two hub genes. We also plotted a calibration curve for this model (Figure 7C), suggesting that the predictive probability of this diagnostic model is close to that of the ideal model. We further plotted a DCA curve for the model (Figure 7D), where the “complex” curve represents the significance of the diagnostic model for guiding clinical decisions. The net benefit of the complex curve was higher than both extreme cases over a wide range of threshold probabilities and higher than when the diagnosis was based on KIAA0319 and PRDX4 alone. This suggests that the model is a good guide for clinical decision-making. The expression of KIAA0319 was significantly elevated in the lacrimal gland of TED patients while PRDX4 was significantly reduced (*p* = 3.9e−06; *p* = 2.9e−05, Figures 7H, I). Orbital adipose tissue enlargement is a prominent pathological manifestation in patients with TED. To identify whether KIAA0319 and PRDX4 are also involved in the process of orbital adipose tissue pathological changes, we also analyzed the difference of orbital adipose tissue samples between TED patients and normal subjects in GSE58331 and compared the expression of KIAA0319 and PRDX4 in the two groups. We found that the difference in the expression of KIAA0319 and PRDX4 between the two groups was not statistically significant (*p* = 0.16; *p* = 0.057, Figures 7J, K). The diagnostic model constructed with KIAA0319, PRDX4 and gender in orbital adipose tissue was not potent in identifying TED (AUC = 0.660, Figure 7G). This suggests that KIAA0319 and PRDX4 may not play a key role in the pathology of orbital adipose tissue in patients with TED.

### 3.7 Functional enrichment analysis of 2 hub genes by single-gene GSEA

**FIGURE 8:**
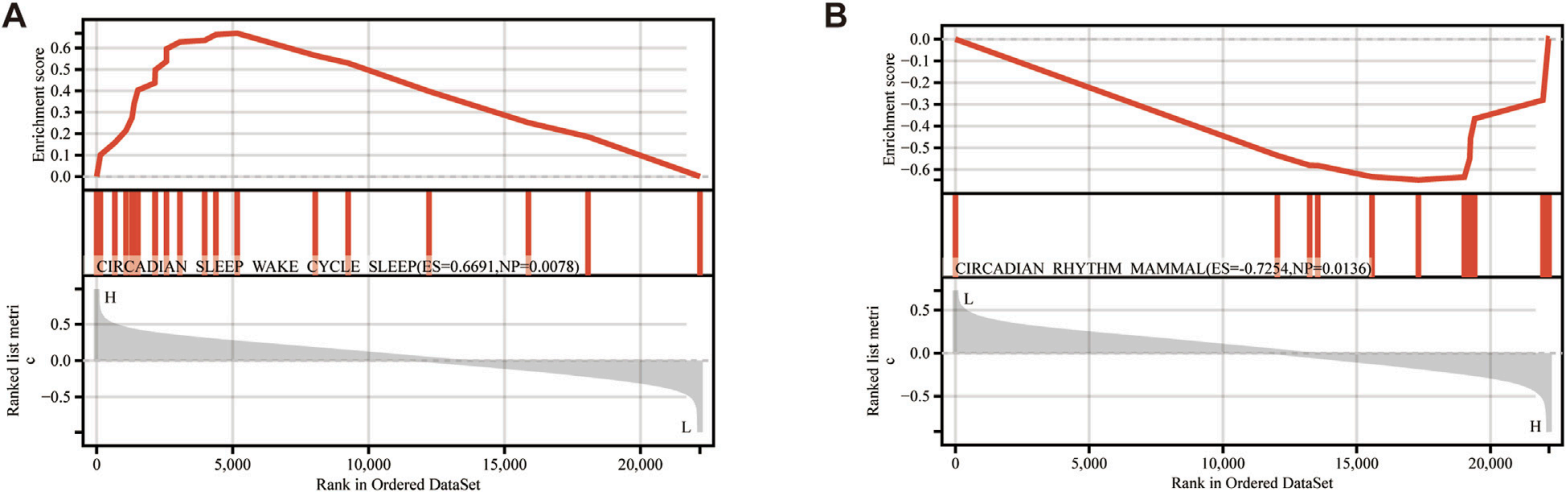
Single gene GSEA. **(A)** GSEA results of KIAA0319. **(B)** GSEA results of PRDX4.

Single-gene GSEA analysis on the TED dataset identified 249 pathways as targets of KIAA0319 and 16 pathways as targets of PRDX4. “Circadian rhythm” related pathways are common among all the pathways enriched for two genes (Figures 8A, B).

### 3.8 Immune cell infiltration analysis

**FIGURE 9:**
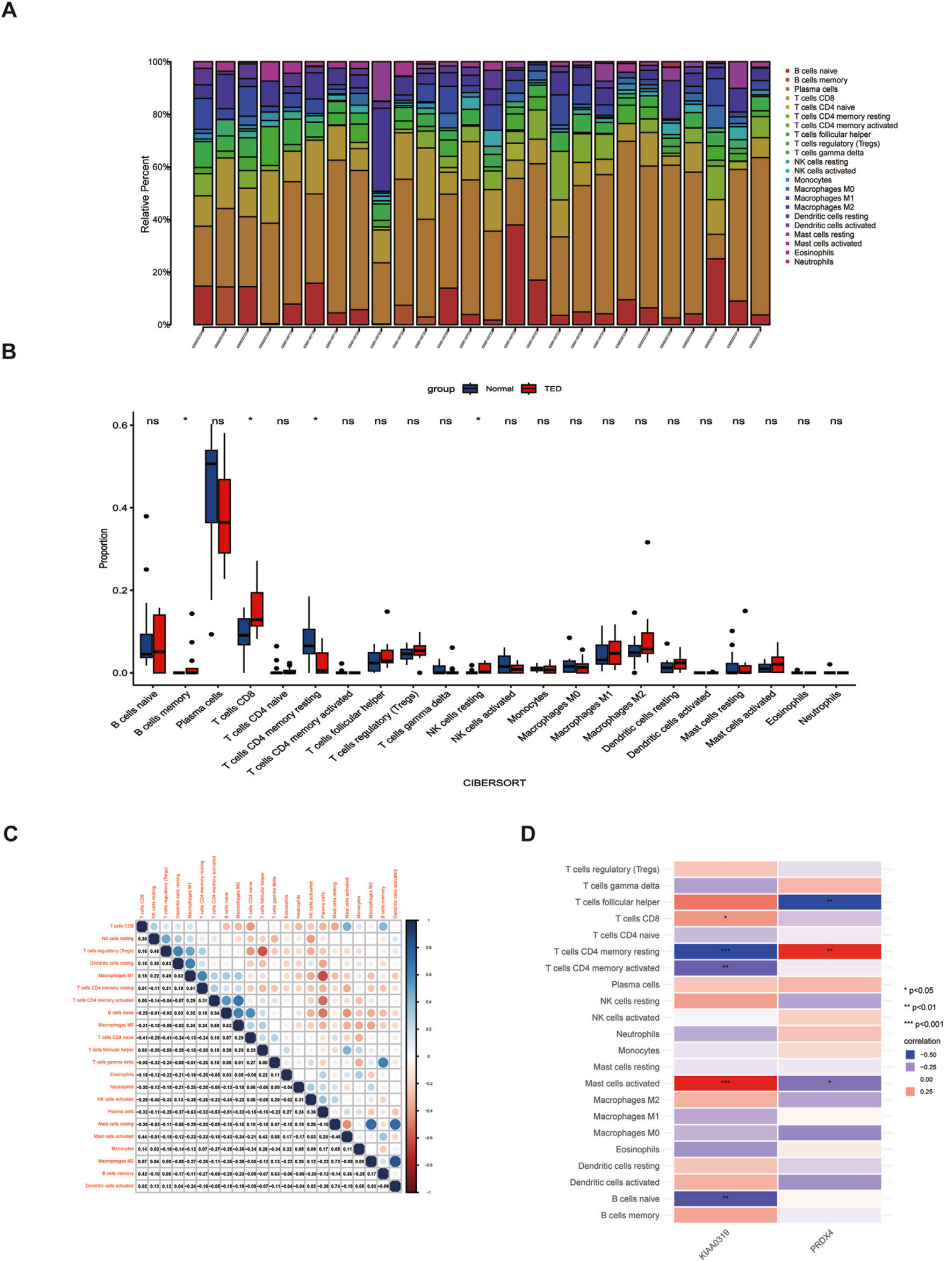
Analysis of immune cell infiltration in the lacrimal gland of TED. **(A)** Histogram displaying the immune cell proportion of the TED and the control group. **(B)** Box plot comparing the infiltration of 22 immune cell types between the TED and control groups. **(C)** Heatmap demonstrating the correlation between 22 immune cell types. **(D)** Association between differentially infiltrated immune cells in TED and the two hub genes. TED: thyroid eye disease.

Immune cells play vital roles in TED pathogenesis (Shu et al., 2024). Previous enrichment analysis of 84 lacrimal gland-associated key genes found that Th1 and Th2 cell differences may participate in the TED-associated lacrimal gland enlargement (Figure 4D). The “Cibersort” method was used to analyze the immune infiltration of the lacrimal gland in the integrated dataset of GSE58331 and GSE105149. The proportion of 22 immune cells in each lacrimal gland sample was analyzed (Figure 9A). In the lacrimal gland of TED patients, the infiltration of memory B cells and CD8+T cells was elevated, while the infiltration of CD4^+^ resting memory T cells and resting NK cells was reduced (Figure 9B). Figure 9C demonstrates the correlation between 22 immune cells. Furthermore, we analyzed the relationship between two hub genes and immune cells. KIAA0319 was positively correlated with the infiltration of CD8+T cells and activated mast cells and negatively with CD4^+^ resting memory T cells, CD4^+^ activated memory T cells and naïve B cells. PRDX4 was positively correlated with the infiltration of CD4^+^ resting memory T cells and negatively with follicular helper T cells and activated mast cells (Figure 9D).

## 4 Discussion

TED is an autoimmune orbital inflammatory disease, whose occurrence is relatively low. However, it seriously affects the QoL of patients and even causes psychological disorders. TED may lead to the aqueous-deficient DED attributed to the lacrimal gland lesions and reduce the score of the QoL. In severe cases, corneal perforation and compressive optic neuropathy can occur and even lead to eyesight loss. Early diagnosis and intervention are essential for TED patients to control orbital inflammation and improve prognosis. However, the diagnosis of TED is highly dependent on the specialized ophthalmologists. Meanwhile, the mechanism underlying the TED-associated lacrimal gland pathological change is still obscure, which hinders the discovery of novel treatments. This study aims to explore the in-depth mechanism of TED-associated lacrimal gland lesions and uncover the potent and non-invasive diagnostic biomarkers in tears.

In this study, we first combined two datasets to obtain an integrated dataset by background correction and normalization. Then, 84 lacrimal gland-associated key genes were obtained by intersecting DEGs and key gene modules, which were obtained by differential gene expression analysis and WGCNA, respectively. To further screen for promising biomarkers in tears, 84 lacrimal gland-associated key genes intersected with the secreted proteins, and 13 LaSGs were identified. GO and KEGG analysis and GGI network analysis all revealed that these 84 genes and the subsequent 13 genes were enriched in endoplasmic reticulum-related pathways. This indicated the disorganization and the dysfunction of the endoplasmic reticulum of the lacrimal gland involved in the lacrimal gland lesions of TED patients. Proteins in tears are secreted primarily by the lacrimal gland acinar cells and secretory proteins are synthesized in the endoplasmic reticulum (Dartt, 1989). The disorganization of the lacrimal gland, indicated by the GO analysis, further encouraged us to explore the secretory proteins, both for their functions in the lacrimal gland pathological changes and their diagnostic significance.

TED is an autoimmune disease. The loss of self-tolerance to TSHR and the production of TRAb are thought to be the core processes of the TED pathophysiology (Smith and Hegedus, 2016). TRAb secreted by plasma cells can bind to the OFs in the orbital adipose tissue and the extraocular muscles and induce the differentiation of OFs into adipocytes and myofibroblasts. Intriguingly, GO-MF analysis of 84 genes demonstrated the enrichment of the thyroid hormone receptor binding pathway, suggesting that TSHR and TRAb may also be the culprits of TED-associated lacrimal gland enlargement mimicking what they are in the orbital connective tissue. This is consistent with the previous study which discovered the expression of the TSHR in the lacrimal gland of healthy people by immunochemistry (Eckstein et al., 2004).

KEGG analysis of 84 genes showed the enrichment of “Fc epsilon RI signaling” and “Th1 and Th2 cell differentiation” pathways. The enrichment of the “Fc epsilon RI signaling” pathway suggested the involvement of Ig (Immunoglobin) E, which is thought to play a role not only in allergy but also in the normal immune responses, antigen processing and presentation (McDonnell et al., 2023), in the pathogenesis of TED-associated lacrimal gland lesions. Though IgG is the main isotype that targets TSHR (Kahaly et al., 2023), IgA and IgE isotypes are also detectable (Metcalfe et al., 2002). The elevated circulating IgE levels are associated with active inflammatory eye signs in TED patients (Molnar et al., 1996). These studies may collectively offer a novel insight into the pathogenesis of the lacrimal gland lesions of TED patients, which may be distinct from the pathogenesis of the orbital adipose tissue enlargement. Th1 and Th2 cell imbalance has been reported by several studies focusing on the immunological regulations of TED and these two cells may participate in the different periods of the TED pathology (Xia et al., 2006; Fang et al., 2021). Whether the lacrimal gland replicates this process needs further research.

Subsequent analysis of immune cell infiltration has demonstrated that there are distinct alterations in the composition of various immune cell subtypes within TED lacrimal samples, in contrast to the control group. An elevated presence of CD8^+^ T cells potentially suggests an active inflammatory state and immune response, which highlights the essential role of the immune system in the pathogenesis of TED-associated lacrimal gland lesions. When comparing the immune infiltration of lacrimal samples and adipose tissue samples of TED and the control group, different infiltration modes can be identified, further indicating the distinct pathological mechanism of two different tissues (Supplementary Figure S1).

To further investigate the significant diagnostic biomarkers in tears, we used four machine-learning algorithms and finally identified KIAA0319 and PRDX4 as the two hub genes. KIAA0319 has been identified as the main candidate dyslexia associated gene though knowledge about it is still limiting. KIAA0319 may support brain development by promoting neuronal migration and regulating the cell cycle during neuroepithelial cell development (Franquinho et al., 2017; Paniagua et al., 2022). Except for the brain, KIAA0319 is also expressed in the eye, sensory and spinal cord neurons (Franquinho et al., 2017; Gostic et al., 2019). No studies were focusing on the function of KIAA0319 in the human lacrimal gland to our knowledge, but it has long been established that the composition of the aqueous layer of the tear film is under tight neural control (Dartt, 2009). Denervation of the rat lacrimal gland altered the protein profile in tears (Hegarty et al., 2018). Nerve abnormality can be observed in the aquaporin 5 (AQP5)-deficiency-induced DED model and the disruption of the axon guidance is regulated by upregulated Slit2 (Bai et al., 2023) which also mediates the orbital fibrocytes differentiation into fibroblasts in TED pathogenesis (Fernando et al., 2021). Whether KIAA0319 influences the TED-associated DED through neural control needs further investigation.

PRDX4, known as peroxiredoxin4, localizes in the endoplasmic reticulum and is characterized by coupling hydrogen peroxide (H_2_O_2_) catabolism with oxidative protein folding (Zito, 2013). It serves as an antioxidant in cells through various mechanisms, including restricting cellular ROS levels, inhibiting ferroptosis (Luo et al., 2022), and reducing oxidative stress and local inflammation (Yamaguchi et al., 2021). Interestingly, in addition to the endoplasmic reticulum, it also co-localizes with inflammasome components within extracellular vesicles (EVs) derived from inflammasome-activated macrophages, thereby restricting inflammasome-mediated signaling by limiting caspase-1 activation (Lipinski et al., 2019). The downregulation of PRDX4 in the lacrimal gland of TED patients indicates that the accumulation of ROS and excessive local inflammation may participate in the lacrimal gland enlargement of TED patients.

We further constructed a nomogram based on the gender and the screened hub genes KIAA0319 and PRDX4. TED patients are predominantly female, so gender was introduced into the model. Analyses including the ROC, DCA, and calibration curve all indicate that the nomogram possesses substantial diagnostic efficacy for patients with TED.

Nowadays, the combination of fluorescence-conjugated contact lenses and mobile fluorescence reading and quantification devices has made tear monitoring at home possible, which may make tear testing an alternative way for disease monitoring (Shi et al., 2022). Compared to blood collection, tear collection is more non-invasive and less dependent on specialized personnel, thus facilitating real-time, continuous monitoring of disease. However, if tear samples are analyzed individually, multiple other causes of dry eye and TED may be confused owing to the complexity of the origin of tear fluid. Therefore, in this study, we selected diagnostic biomarkers by intersecting pathologically significant genes in TED-associated lacrimal gland lesions and secretory proteins to improve the specificity.

While the findings of this study offer significant insights that are poised to inform future clinical practice, it is imperative that these discoveries be substantiated through further experimental validation and comprehensive clinical research to establish a more robust scientific foundation. A large number of tear fluid samples from TED patients can be collected for the diagnostic model validation. ELISA can be applied for testing KIAA0319 and PRDX4 in the tear fluid. Fluorescence-conjugated contact lenses and other developing tear-monitoring devices may provide additional methods for convenient and efficient tear monitoring. Moreover, this research has primarily centered on the diagnosis of TED; however, there remains a critical need for the development of effective treatment strategies and preventive interventions for TED-associated lacrimal gland lesions.

In summary, this comprehensive investigation delves into the mechanisms underlying TED-associated lacrimal gland lesions through multifaceted analysis. It underscores the pivotal role of endoplasmic reticulum disorganization in TED-associated lacrimal gland lesions and introduces a novel diagnostic model based on the KIAA0319/PRDX4 biomarkers. The contributions of this study not only enhance our understanding but also provide vital scientific evidence for advancing clinical practices.

## Data Availability

Publicly available datasets were analyzed in this study. This data can be found here: GEO data repository, accession numbers as follows: GSE58331, GSE105149.
