# Supplementary figures and images for: Screening of pathologically significant diagnostic biomarkers in tears of thyroid eye disease based on bioinformatic analysis and machine learning
Source: Front Cell Dev Biol. 2024 Oct 30;12:1486170. doi: 10.3389/fcell.2024.1486170 (PMC11561714; doi:10.3389/fcell.2024.1486170)

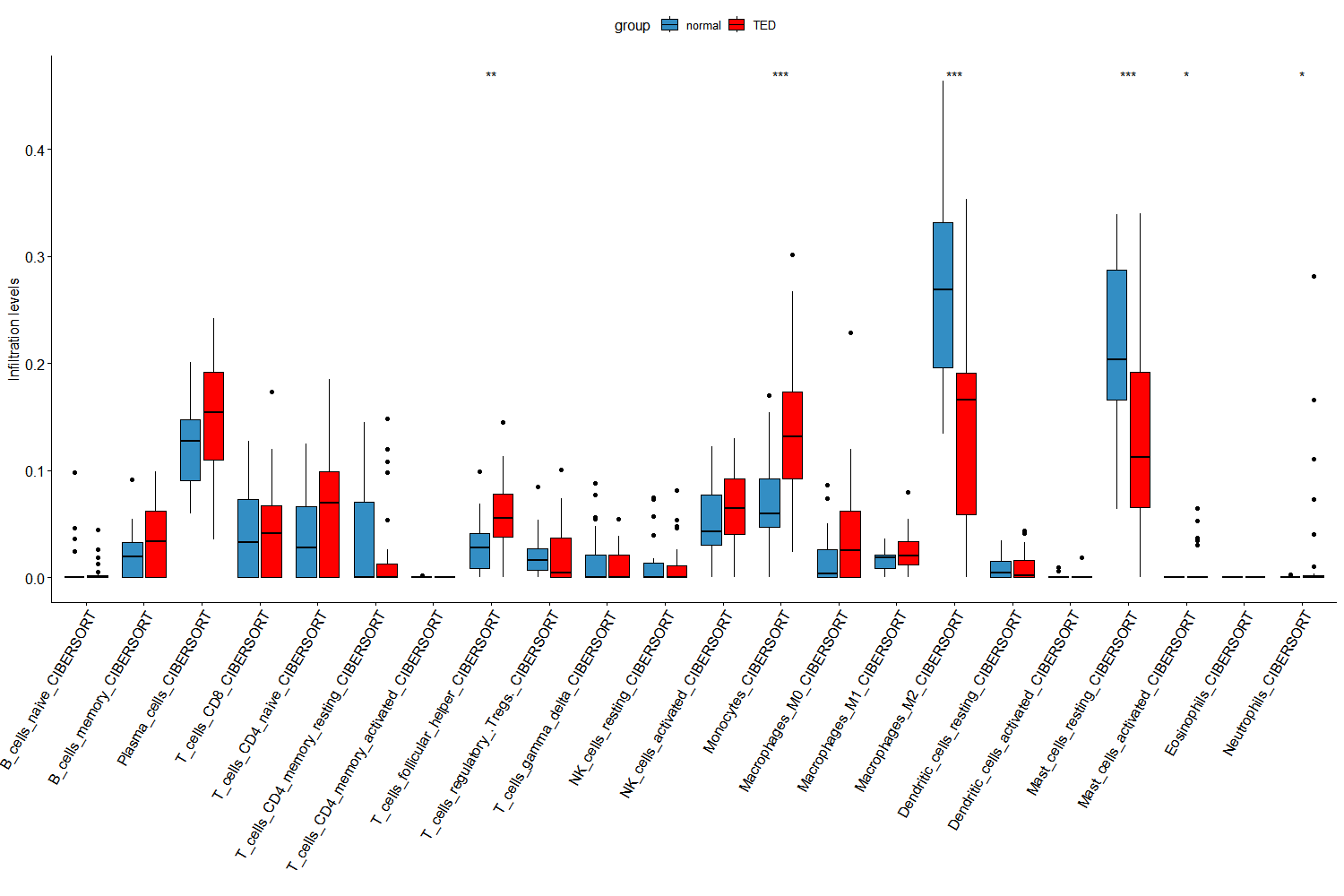

Supplement: Supplementary file 1 [file Image1.TIFF]
